# Supplementary material for: A combination of plasma membrane sterol biosynthesis and autophagy is required for shade-induced hypocotyl elongation
Source: Nat Commun. 2022 Oct 10;13:5659. doi: 10.1038/s41467-022-33384-9 (PMC9550796; doi:10.1038/s41467-022-33384-9)
Supplement: Supplementary file 1 — Supplementary Information [file 41467_2022_33384_MOESM1_ESM.pdf]

Supplementary Information

A combination of plasma membrane sterol biosynthesis and autophagy is required for shade-induced hypocotyl elongation.

Yetkin Çaka Ince [1], Johanna Krahmer [1], Anne Sophie Fiorucci [1], Martine Trevisan [1], Vinicius Costa Galvão [1], Leonore Wigger [2], Sylvain Pradervand [2], Laeticia Fouillen [3], Pierre Van Delft [3], Manon Genva [3], Sebastien Mongrand [3], Hector Gallart-Ayala [4], Julijana Ivanisevic [4] and Christian Fankhauser [1*]

Supplementary figures 1 to 9

Supplementary table 1 and 2

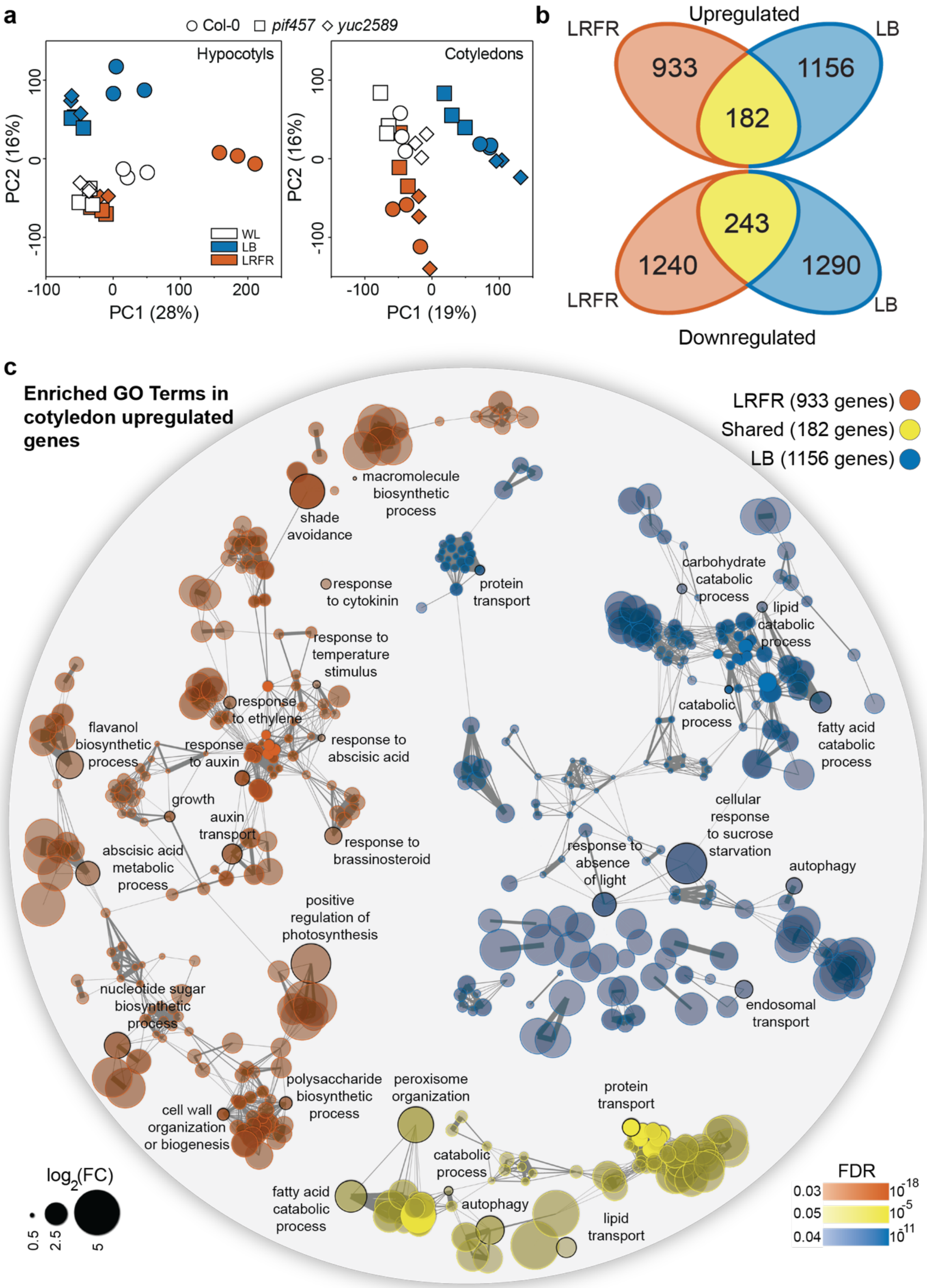

Supplementary Figure 1. LB and LRFR induce distinct transcriptional changes in cotyledons. (a) Principal component (PC) analysis of hypocotyl and cotyledon transcriptomes. PC1 and PC2 of each biological replicate (n = 3) are graphically visualized. (b) Number of up- and downregulated genes in Col-0 cotyledons in the indicated light

conditions (FDR<0.05, T-test with BH correction). (c) GO term enrichment analysis in the cotyledon upregulated gene lists. Each node indicates a significantly enriched GO term (FDR<0.05). Two terms (nodes) are connected if they share 20% or more genes. The line thickness increases with the increased number of shared gene sets between two terms. Only selected GO terms (black circles) are annotated. The full list of enriched GO terms is in Supplementary data 2, the interactive version of (c) is available https://figshare.com/s/268fb1b265883e46f671 See also Figure 1.

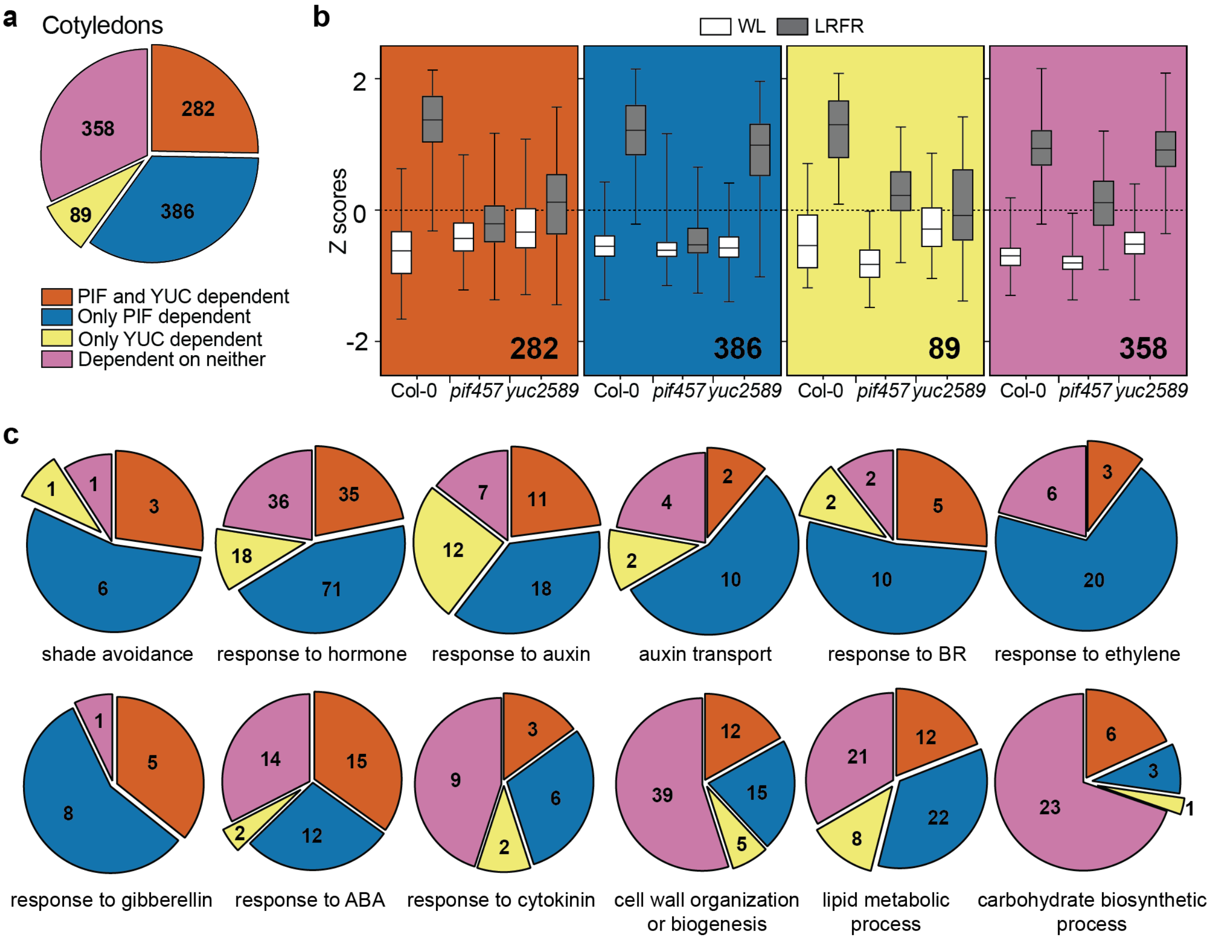

Supplementary Figure 2. PIFs & YUCs are required for hormone-associated transcriptional changes in LRFR in cotyledons.

(a) The distribution of cotyledon-induced genes in LRFR according to the dependence on PIFs and YUCs using the comparison of Col-0, *pif457* and *yuc2589* transcriptomes (FDR<0.05, F-test with post-hoc test). (b) Distributions of Z- scores computed from replicates averages for categories shown in (a). The horizontal bar represents the median; boxes extend from the 25th to the 75th percentile, whiskers extend to show the data range. (c) The distribution of cotyledon-induced genes according to the dependence on PIFs and YUCs in each of the selected significantly enriched GO terms. Numbers indicate significantly regulated genes in the given categories and/or GO terms. The full list of misregulated genes and enriched GO terms are given in Supplementary Data 3. See also Figure 2.

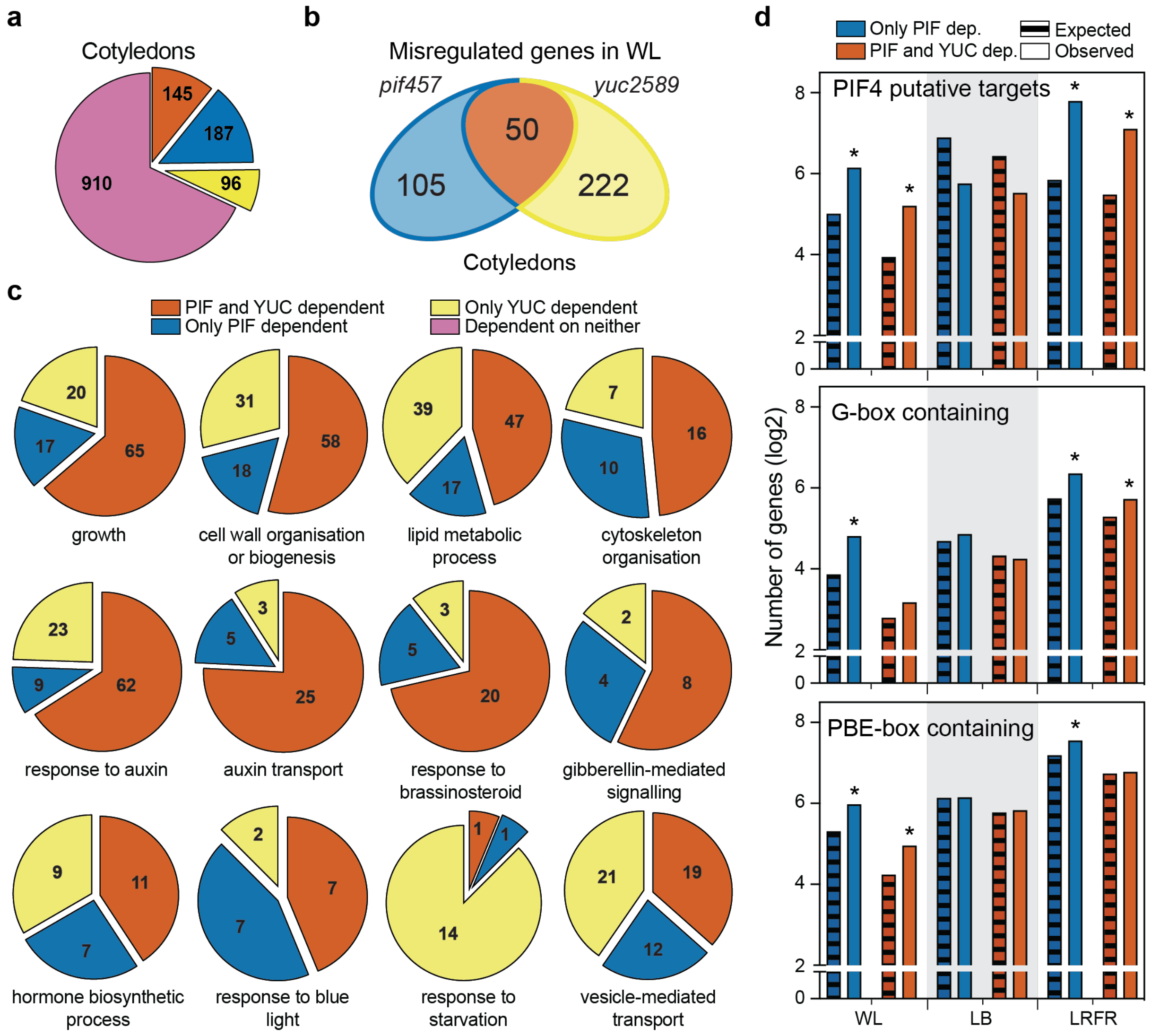

Supplementary Figure 3. PIFs & YUCs are required for basal expression of many growth- and hormone- associated genes in WL in cotyledons.

(a) The distribution of LB-induced genes in cotyledons according to the dependence on PIFs and YUCs using the comparison of Col-0, *pif457* and *yuc2589* transcriptomes (FDR<0.05, F-test with post-hoc test). (b) Number of misregulated genes in *pif457* and *yuc2589* hypocotyls compared to Col-0 in WL (FDR<0.05, T-test with BH correction). (c) The distribution of misregulated genes in *pif457* and *yuc2589* hypocotyls (as grouped in Fig. 3C) in each of the selected significantly enriched GO terms. The full list of enriched GO terms is given in Supplementary Data 5. (d) Comparison of PIF dependent genes in cotyledons with putative PIF4 targets (as listed in 26), promoters (1 kb upstream) containing G-box (CACGTG) or PBE-box (CATGTG). Asterisks (*) indicate statistically significant overrepresentation compared to expected (P < 0.05, Binomial distribution, one tailed; the exact P values are available in the Source Data). Numbers indicate significantly

regulated genes in the given categories and/or GO terms. The full list of misregulated genes, enriched GO terms, and enriched motifs are given in Supplementary Data 4, 5, and 6. See also Figure 3.

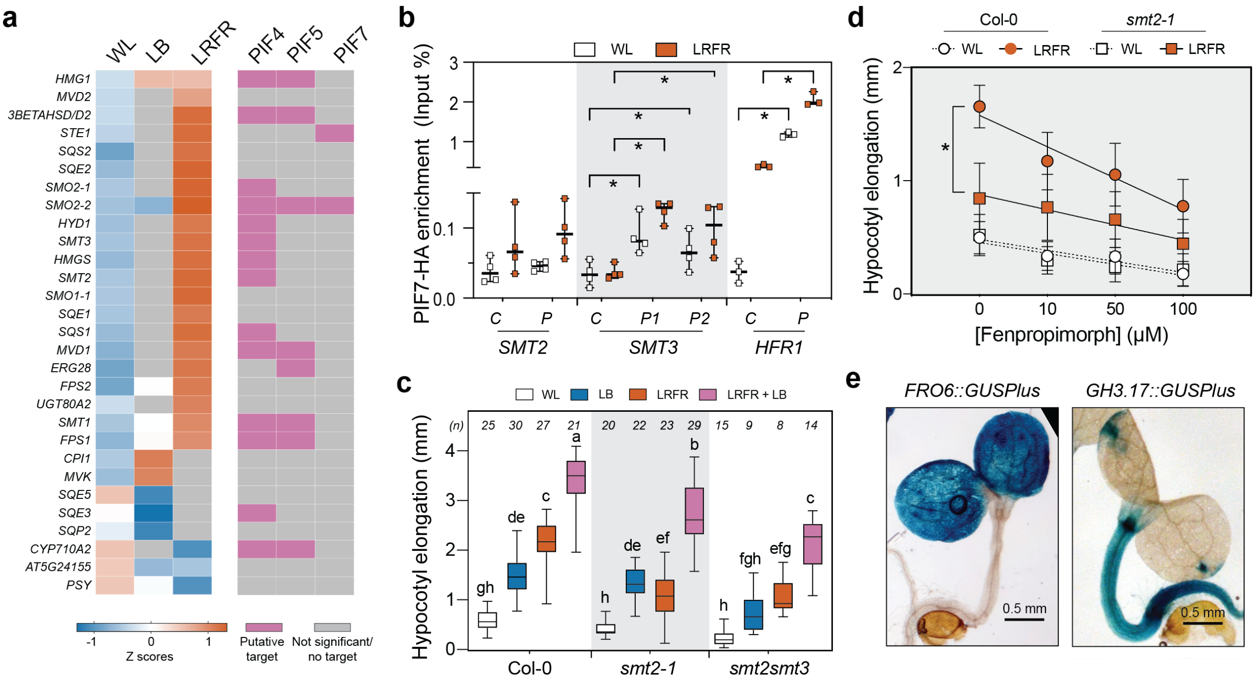

Supplementary Figure 4. SMT2 is locally required for LRFR-induced hypocotyl elongation.

(a) The heatmap showing average Z-scores of genes annotated in sterol biosynthesis (GO: 0016126) in RNA-seq and putative binding of PIF4, PIF5 and PIF7 (ChIP-seq data from [26,45]). A gene is shown if it is significantly regulated at least in one treatment (LB or LRFR). (b) PIF7-HA binding to the promoter of the indicated genes. PIF7-HA enrichment is quantified by qPCR and presented as IP/Input. Each data point indicates technically independent samples, horizontal bar represents the median, whiskers extend to show the data range. Control – C, Peak – P. (c, d) Hypocotyl elongation of indicated genotypes in the indicated conditions. (c) The horizontal bar represents the median; boxes extend from the 25th to the 75th percentile, whiskers extend to show the data range. (b, d) Data are means ± SD with a regression line. Different letters (C, two-way ANOVA with Tukey's HSD test) and asterisks (*) (b, Student's T-test, one-tailed) (d, two-way ANOVA) indicate significant difference ($P < 0.05$) compared to control (b), and between genotypes in given light condition (d). The exact P values are available in the Source Data. Sample size (n) that is given on top (c); ((d) For Col-0: n (WL) = 17, 16, 19, 19; n (LRFR) = 21, 21, 15, 20. For *smt2-1*: n (WL)= 15, 21, 18, 14; n (LRFR) = 16, 20, 15, 19 with an order of Mock, 5 µM, 50 µM, 100 µM respectively) indicates biologically independent seedlings examined over one experiment. The experiments were repeated three (c) and two (d) times with similar results. (e) GUS staining of the indicated *promoter::GUSPlus* lines. See also Figure 4.

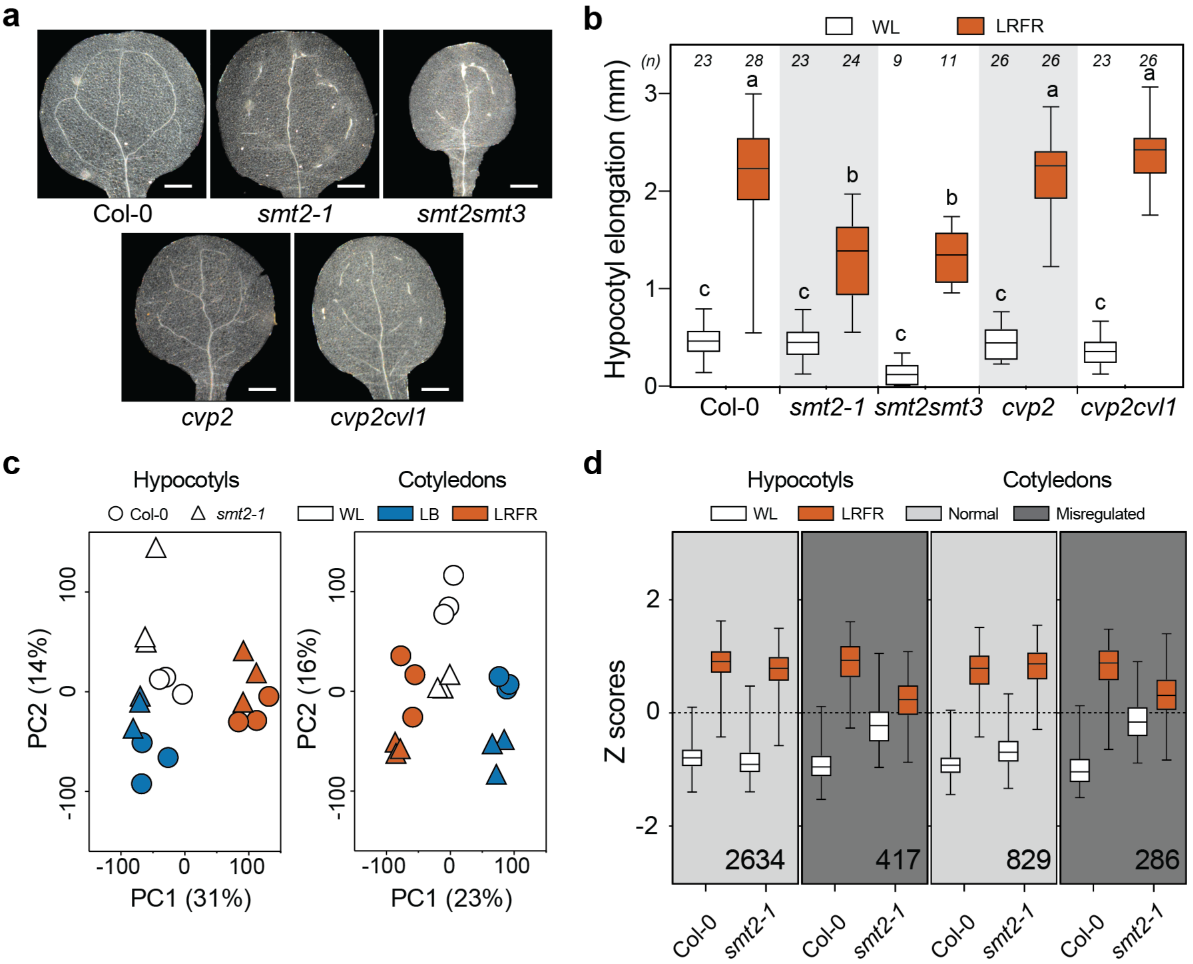

Supplementary Figure 5. Phenotyping evaluation of *cotyledon vasculature pattern* (*cvp*) mutants and *smt2-1* transcriptome summary.

(a) Representative images of cotyledon vasculature phenotype of indicated genotypes. White bars equal to 200 µm. (b) Hypocotyl elongation of indicated genotypes. Different letters indicate significant difference ($P < 0.05$, two-way ANOVA with Tukey's HSD test; the exact P values are available in the Source Data). Sample size (n) that is given on top indicates biologically independent seedlings examined over one experiment. The experiment was repeated three times with similar results. (c) PCA of Col-0 and *smt2-1* transcriptomes. PC1 and PC2 of each biological replicate ($n = 3$) are graphically visualized. (d) Distributions of Z- scores computed from replicates averages for LRFR-induced genes that are grouped comparing Col-0 and *smt2-1* transcriptomes (FDR<0.05). 'Normal' and 'Misregulated' refer to expression in *smt2-1*. The numbers indicate significantly regulated genes in each group (FDR<0.05, F-test with post-hoc test). (b, d) The horizontal bar represents the median; boxes extend from the 25th to the 75th percentile, whiskers extend to show the data range. The full list of misregulated genes is given in Supplementary Data 7. See also Figure 5.

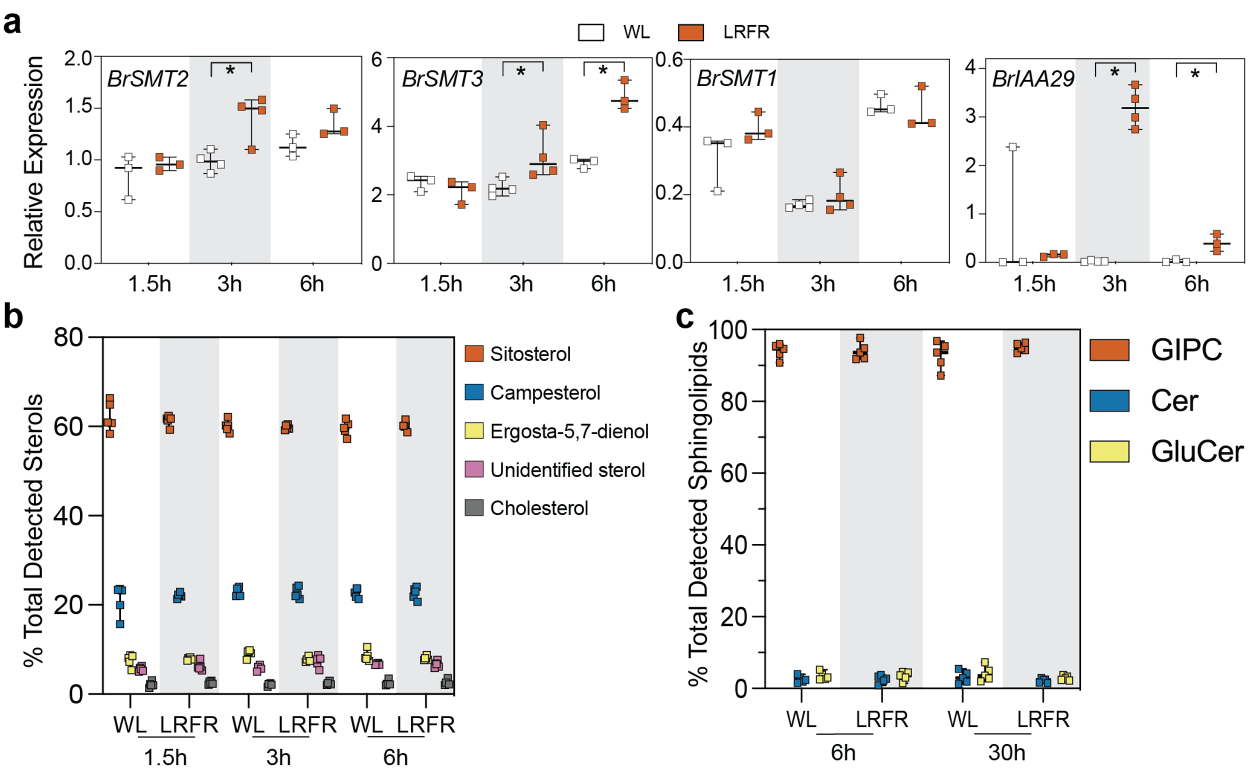

Supplementary Figure 6. Sterol and Sphingolipid composition of *B. rapa* hypocotyls does not change in LRFR

(a) Relative expression of the indicated genes in 5-d-old *B. rapa* hypocotyls. Gene expression values were calculated as fold induction relative to *BrPP2A*. n = 4 (biological) with three technical replicates for each RNA sample. Asterisks (*) indicate P < 0.05 (Student's T-test, one-tailed, the exact P values are available in the Source Data). Sterol (b) and Sphingolipid (c) composition of 5-d-old *B. rapa* hypocotyls. GIPC, Glycosyl Inositol Phospho Ceramides; Cer, Cermides; GluCer, Glucosylceramide. (a, b, c) Each data point indicates biologically independent samples, horizontal bar represents the median, whiskers extend to show the data range.. See also Figure 6.

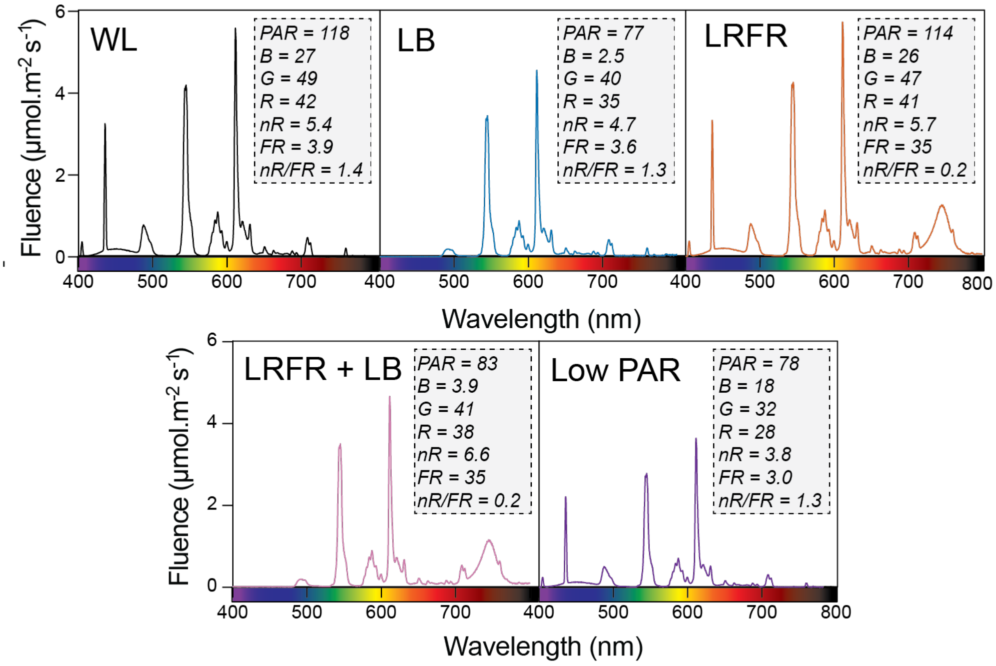

Supplementary Figure 7. Light conditions used in this study.

Photosynthetically active radiation (PAR, 400-700 nm), Blue (B, 400-500 nm), Green (G, 500-600 nm), Red (600-700 nm), Narrow R (nR, 640-700) and far-red (FR, 700-760 nm).

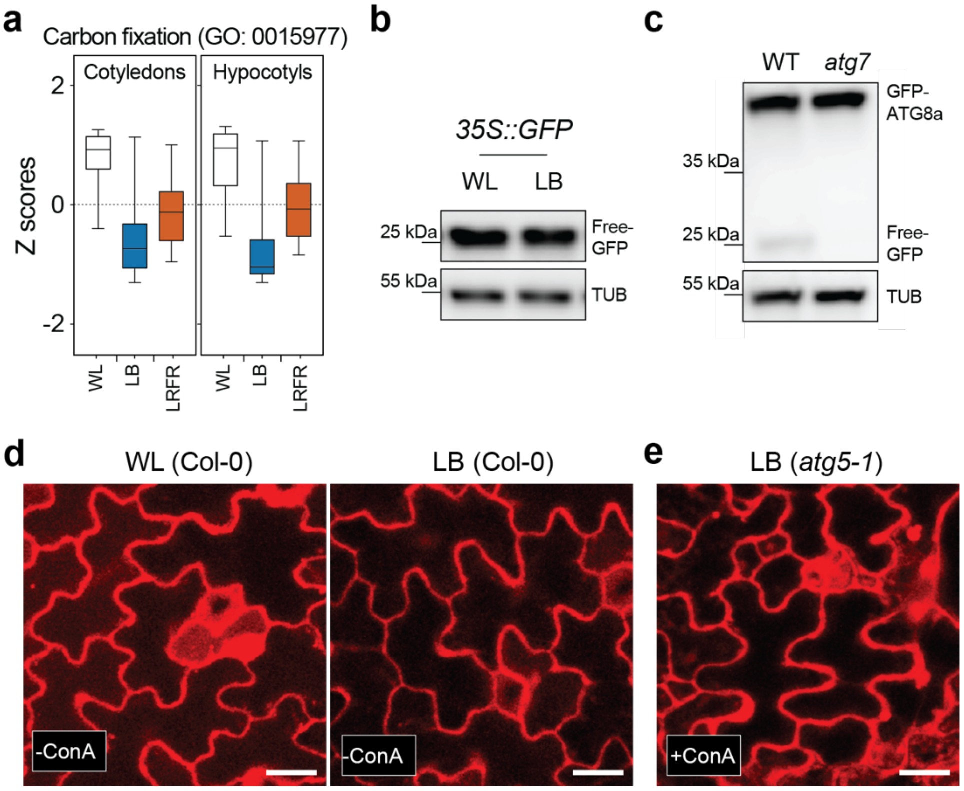

Supplementary Figure 8. Induction of autophagy depends on LB and the autophagy pathway.

(a) Distributions of Z- scores computed from replicates averages for genes listed in carbon fixation GO term in Col-0 seedlings. The horizontal bar represents the median; boxes extend from the 25th to the 75th percentile, whiskers extend to show the data range. The full list of genes in Carbon fixation GO term is given in Supplementary Data 10. (b) Western blot assay using a *35S::GFP* line grown in WL with and without 8h LB. (c) Western blot assay of GFP-ATG8a and Free-GFP levels in WT and *atg7-2* seedlings treated with 8h of LB in the presence of 0.5 µM ConA. In (b) and (c) TUB was used as a loading control. The experiments were repeated four times with similar results. (d, e) Representative image of cotyledon pavement cells of *UBQ10::mCherry-ATG8e* (d) in Col-0 kept in WL or treated with LB in the absence of ConA for 8h and (e) in *atg5-1* treated with LB in the presence of ConA for 8h. The experiment was repeated two times with similar results using 5 to 7 biologically individual seedlings for each condition and the experiment. White bars equal 20 µm. See also Figure 7.

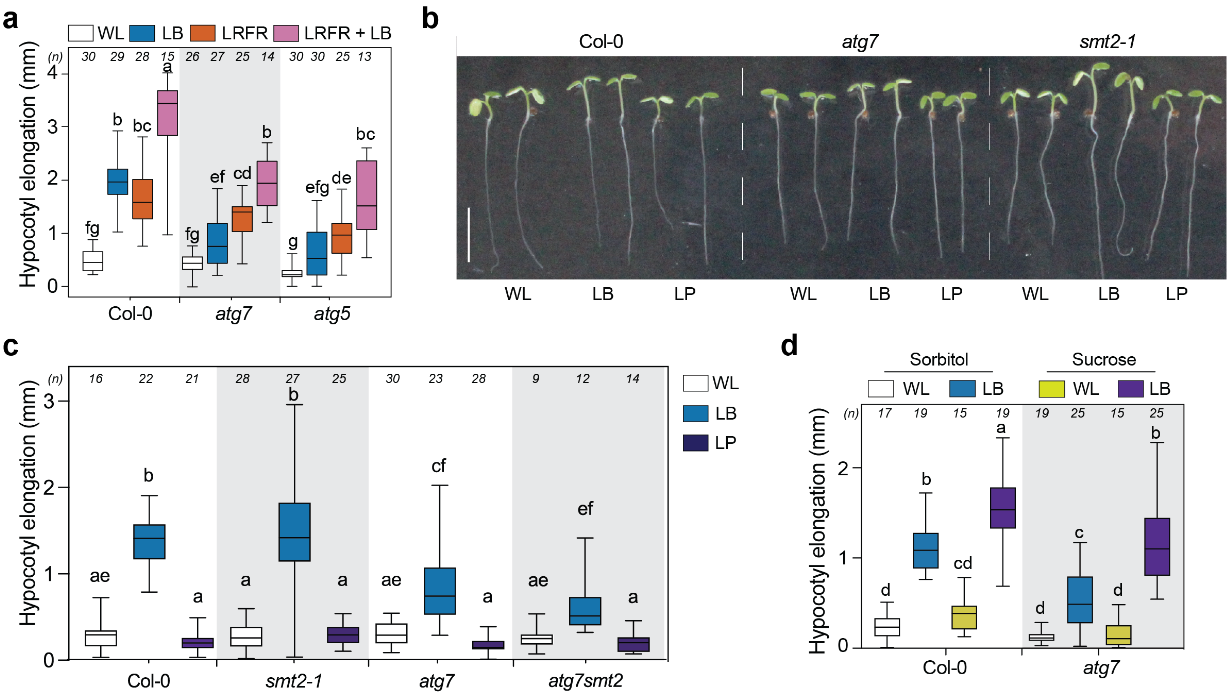

Supplementary Figure 9. Regulation of hypocotyl elongation in autophagy mutants.
(a) Hypocotyl elongation of the *atg* mutants in LB, LRFR and LRFR+LB. (b) Representative pictures of the indicated genotypes in the indicated light conditions. White bar equals to 5 mm. (c) Hypocotyl elongation of *smt2*, *atg7* and *smt2atg7* in WL, LB and LP. Col-0 data is the same as the one shown in Figure 8c (left panel). (d) Hypocotyl elongation of the indicated genotypes in WL and LB with or without external sucrose. (a, c, d) The horizontal bar represents the median; boxes extend from the 25th to the 75th percentile, whiskers extend to show the data range. Different letters indicate significant difference ($P < 0.05$, two-way ANOVA with Tukey's HSD test; the exact P values are available in the Source Data). The experiments were repeated three (a) and two (c, d) times with similar results. See also Figure 8.

Supplementary Table 1: List of oligonucleotides used in the study

**Included She**	**Description**
Genotyping	List of oligonucleotides used in genotyping
Cloning	List of oligonucleotides used in cloning
ChIP-qPCR	List of oligonucleotides used in ChIP-qPCR
RT-qPCR	List of oligonucleotides used in RT-qPCR

**Allele**	**Collection**	**Name**	**Sequence**
		YI428	TCACCATCAATCCCCGGAAA
		YI429	CTGCGTACACTTCTTCCAGC
smt2-1 (frl1)	EMS*	*PCR is followed by digestion with BsaHI	
		YI426	CTCTCTCTTGGTCTTCCTCACTCTTCACGAAAAT
		YI427	AACCAGTAGATACCGACGGCGAC
smt2-2 (cvp1-3)	EMS**	**PCR is followed by digestion with MslI	
		YI430	CATGATTTTATTTTGTGAAGAAAAATG
smt3-1	SALK_085292	YI431	CCAGCTTTCTTGTTGTGAAGC
		OM181	CGG TGT ATC CAC GGG AGT AA
		OM182	CGC TTG TTG AGA TGC AGA AT
cvp2-1	EMS***	***PCR is followed by digestion with BslI	
		OM183	TGA TCA GAA AAC CGT GAC TCC
cvl1-1	SALK_029945	OM184	AGC ACA TTT TTG AAT TCA CCG
		MT458	GAATCCAGCCGCGTAAAGTC
yuc5-1	SAIL_116_CO1	AG178	GACGAGACAAGTGGTCTCTGG
		YI444	GGAGCTTAACAAAGGGAAACG
atg7-2	GABI_655B06	YI445	CGTGTAACAGTGCATTGTTGG
		oJM1845	TTCTTGCATTTTCTCGCTCTACG
yuc2-1	SALK_030199	MT440	AACCCGTGGCGAGTATAATG
		oJM1206	CATCCTCTCCACGTGGCTTCC
yuc8-1	CS110939	oJM1207	GAACTGACGCTTCGTCGGGTAC
		oJM1199	GCTCGGTAAGCAAAACAAAACTG
yuc9-1	SAIL_871G01	oJM1200	GAAGGAAATGCCCAATGAGAC
		SL-43	CAGACGGTTGATCATCTG
pif4-101	SAIL_114_G06	oVCG-61	TAGCATCTGAATTTCATAACCAATCTCGATACAC
		SL-46	TCGCTCACTCGCTTACTTAC
pif5-3 (pil6-1)	SALK_087012	oVCG-56	ATTTTGCCGATTTCGGAAC
		SL-195	GTGGCAAGTTGGCTCTTAGG
pif7-1	CS68809	SL-169	TGATAGTGACCTTAGGCGACTTTTGAACGC

		oASF-27	GGAGAGCCATAGAGTTGG
pif7-2	SAIL_622_G02	oVCG-61	TAGCATCTGAATTTCATAACCAATCTCGATACAC

Plasmid	Target	Name	Sequence
pYI001			
(GUSPlus::tOCS in pFP100)*		YI001	TCAAGCTAAGCTTGCATGCCCGGGAAAATGGTAGATCTGAGGGTAA
	GUSPlus::tOCS	YI002	CCTAAAACCAAAATCCAGTGGCGCTCTAGAGGTCCTGCTGA
pYI006 (pFRO6::GUSPlus::tOCS)**		YI011	ATCCAAGCTCAAGCTAAGCTcgatgctctcaaggccaa
	pFRO6	YI012	TAGAAATTTACCCTCAGATCTACCATctttatttgaatttccacttctc
pYI012 (pGH3.17::GUSPlus::tOCS)*		YI020	ATCCAAGCTCAAGCTAAGCTCATAAACATTTACCTTTCATGG
	pGH3.17	YI024	TAGAAATTTACCCTCAGATCTACCATtTtctgaaagcagacacaaacaaagc
pYI046			
(pUBQ10::SMT2-FLAG::tOCS)*		YI052	ATCCAAGCTCAAGCTAAGCTTCGACGAGTCAGTAATAAACGGC
	pUBQ10	YI070	AAGAGTCCATTTTCCGCGGGctagtCTGTT
		YI071	CCCGCGGAAAATGGACTCTTTAACACTCTTCTT
	SMT2-FLAG	YI072	TATCTCATTAAAGCAGGATCCTCACTTGTCATCGTCGTCCTTGTAATCAGAACTCTCCTCCGGT
		YI056	GTGAAGGATCCTGCTTTAATGAGA
	tOCS	YI002	CCTAAAACCAAAATCCAGTGGCGCTCTAGAGGTCCTGCTGA
pYI047			
(pFRO6::SMT2-FLAG::tOCS)**		YI011	ATCCAAGCTCAAGCTAAGCTcgatgctctcaaggccaa
	pFRO6	YI073	AAGAGTCCATctttatttgaatttccacttctcagtgttg
		YI074	tcaaataaagATGGACTCTTTAACACTCTTCTTCACC
	SMT2-FLAG	YI072	TATCTCATTAAAGCAGGATCCTCACTTGTCATCGTCGTCCTTGTAATCAGAACTCTCCTCCGGT
pYI048			
(pGH3.17::SMT2-FLAG::tOCS)**		YI020	ATCCAAGCTCAAGCTAAGCTCATAAACATTTACCTTTCATGG
	pGH3.17	YI075	AAGAGTCCATtTtctgaaagcagacacaaacaaag
		YI076	ctttcagaAaATGGACTCTTTAACACTCTTCTTCACCG
	SMT2-FLAG	YI072	TATCTCATTAAAGCAGGATCCTCACTTGTCATCGTCGTCCTTGTAATCAGAACTCTCCTCCGGT
**Strategy ***	Digestion of pFP100 with KpnI and HindIII was followed by in fusion		
**Strategy *****	Digestion pYI001 with HindIII and BglII was followed by in fusion cloning		

Target	Name	Sequence
SMT2 (peak)	YI633	AGGTCCCTAGAGTGAGGGTG
	YI634	CTCCACCACCACACGTGATT
SMT2 (Contrc	YI522	TACGAGTGGGTTACGACGGA
	YI523	CGCCTCTCTCAATCCCTTGG
SMT3 (Peak 1	YI653	GGTCCTTGGGGTATCCAATTAT
	YI654	GAGTCATGCATGTGATACGACC
SMT3 (Peak 2	YI659	TTTTCCCTGCCTGACCCTTG
	YI660	GCAAAAATGAAGACGGATCATGG
SMT3 (Contrc	YI528	TACCAAGTGCAACGAGCCAA
	YI529	ACGTGTTTTCATCGAACGGC
HFR1 (Peak)	PH112	ACGTGATGCCCTCGTGATGGAC
	PH113	GTCGCTCGCTAAGACACCAAC
HFR1 (Contro	PH126	ACGCAACAAACGAACCACAC
	PH127	AGAGCGATCGGATCAGATAG

Target	Name	Sequence
BrSMT2	YI636	GCCGAGATCTACAGGGTGTT
	YI637	TTGGATGACCTCCACGTGTT
BrSMT3	YI640	GATGGGTCGGATTGCGTACT
	YI641	TCCCAACAGCAGACAGAACC
BrSMT1	YI650	GCCAGTCAGACAAGGAAGAT
	YI651	CCTCGTCGTACACAACAGAA
BrIAA29	YI668	TGCATTTGACCCTGACAACG
	YI669	TGGCCAGATCCTTTTCCCAT
BrPP2A	YI656	AACGCCCCCGATACGAATTA
	YI657	CCACGGTCTACATAGTCACCC

Supplemental Table 2. List of the key resources used in the study

**Reagent or Resource**	**Source**	**Identifier**
**Biological Samples**		
DNA and RNA from various plant tissues from *Arabidopsis* and *B. rapa*	See methods	N/A
Hypocotyl samples from *B. rapa* for lipidomics, sterol, and sphingolipid measurements	See methods	N/A
**Chemicals, peptides, recombinant proteins, and commercial assays**		
Murashige and Skoog medium including vitamins	Duchefa Biochemie, Haarlem, Netherlands	M0222.0010
Phytoagar	Agar-Agar, plant; Roth	Art.-Nr. 4807.3
Sucrose	AppliChem	A2211,1000
Sorbitol	AppliChem	A2222,0500
Fenpropimorph	Carbosynth, United Kingdom,	FF23264
Picloram	Sigma-Aldrich, Steinheim, Germany,	P5575
Concanamycin A	Sigma-Aldrich, Steinheim, Germany,	C9705

Fast SYBR Green Master Mix	Applied Biosystems	Cat# 4385612
Phusion® High-Fidelity DNA Polymerase	New England Biolabs, Massachusetts, USA,	Cat. No. M0530
In Fusion® HD Cloning kit	Takara, California, USA	Cat. No. 639649
RNAlater™-ICE	Thermo Fisher Scientific, United States	AM7030
NEBNext Ultra II Directional RNA Library Prep Kit for Illumina	New England Biolabs, Massachusetts, USA	E7760L
Illumina HiSeq 3000/4000 SR Cluster Kit	Illumina; San Diego, California, USA	TG-410-1005
HiSeq 3000/4000 SBS Kit	Illumina; San Diego, California, USA	GD-410-1001
KpnI	New England Biolabs, Massachusetts, USA,	R0142S
HindIII-HF	New England Biolabs, Massachusetts, USA,	R3104S
BglII	New England Biolabs, Massachusetts, USA,	R0144S

BsaHI	New England Biolabs, Massachusetts, USA,	R0556S
MslI	New England Biolabs, Massachusetts, USA,	R0571S
BslI	New England Biolabs, Massachusetts, USA,	R0555S
Trans-Blot ® Turbo ™ RTA Transfer Kit, Nitrocellulose	Bio-Rad, Hercules, CA, USA	Cat. No. 170-4270
10% Mini-Protean TGX gels	Bio-Rad, Hercules, CA, USA	Cat. No. 4561036
Anti-GFP JL-8	Clontech, California, USA	Cat. No. 632380/632381
Polyclonal H3	Abcam, Cambridge, UK	Cat. No.1791
Anti-TUB	Abicode, California, USA	Cat. No. M0267-1a
Horseradish peroxidase (HRP)-conjugated anti-mouse	Promega, Madison, USA	Cat. No. W4011
Horseradish peroxidase (HRP)-conjugated anti-rabbit	Promega, Madison, USA	Cat. No. W4021
Immobilon® Western	Millipore, Merck KGaA,Darmstadt,	Cat. No. WBKLS0500

Chemiluminescent HRP Substrate	Germany	
BSTFA-trimethylchlorosilane	Sigma-Aldrich, Steinheim, Germany	Cat. No. B-023
SPLASH® LIPIDOMIX® Mass Spec Standard	Sigma-Aldrich, Steinheim, Germany,	330707-1EA
Zorbax Eclipse Plus C18	Agilent technologies, USA	1.8 µm, 100 mm × 2.1 mm I.D. column
Various Oligonucleotides	See Supplementary Table 11	N/A
**Deposited data**		
RNA-seq data	This study	GSE174655
**Experimental models: organisms/strains**		
Agrobacterium tumefaciens GV3301	This study	
Escherichia coli DH5alpha	This study	
Arabidopsis thaliana (Columbia-0)	This study	
smt2-1 (frl1)	[1]	EMS mutant (See STAR methods), provided by D. Gleen
smt2-2 (cvp1-3)	[2]	EMS mutant (See STAR methods), provided by D. Gleen

smt3-1	[3]	SALK_085292, provided by D. Gleen
pif4-101	[4]	SAIL_114_G06
pif5-3 (pil6-1)	[5]	SALK_087012
pif7-1	[6]	CS68809
pif4pif5pif7	[7]	*pif4-101, pif5-3, pif7-1*
pif7-2	[6]	SAIL_622_G02
PIF7-HA line (pif7-2/pPIF7::PIF7-3HA-tPIF7)	[8]	N/A
PIF4-HA line (pif4-101/pPIF4::PIF4-3HA-tPIF4)	[9]	N/A
cvp2-1	[10]	EMS mutant (See STAR methods), provided by C. Hardtke
cvp2-1cvl1-1	[10]	SALK_029945 (cvl1), provided by C. Hardtke
DII-VENUS	[11]	Provided by T. Vernoux
DII-VENUS (in *smt2-1*)	This study	N/A
atg7-2	[12]	GABI_655B06, prov ided by R. Vierstra
smt2atg7	This study	*smt2-1, atg7-2*
atg5-2	[13]	SAIL_129B07, provided by Y.

		Dagdas
35S::ATG8a-GFP	[13]	Provided by R. Vierstra
35S::ATG8a-GFP (atg7-2)	See above	Provided by R. Vierstra
35S::GFP	This study	Provided by L. Allenbach
yuc2-1	[14]	SALK_030199
yuc5-1	This study	SAIL_116_C0
yuc8-1	[14]	CS110939
yuc9-1	[14]	SAIL_871G01
yuc2yuc5yuc8yuc9	This study	yuc2-1, yuc5-1, yuc8-1, yuc9-1
UBQ10::mCherry-ATG8e	[15]	Provided by Y. Dagdas
UBQ10::mCherry-ATG8e (atg5-1)	[16]	Provided by Y. Dagdas
**Recombinant DNA**		
pFP100	[17]	N/A
pYI001	This study	Addgene ID: 170833
pYI006	This study	Addgene ID: 170840
pYI012	This study	Addgene ID: 170841
pYI046	This study	Addgene ID: 170842
pYI047	This study	Addgene ID: 170843
pYI048	This study	Addgene ID: 170844
**Software and algorithms**		
bcl2fastq Conversion Software v.2.20	Illumina; San Diego, California, USA	N/A
Cutadapt (v. 1.8)	[18]	
fastq_screen (v. 0.11.1)	[19]	N/A

eaper (v. 15-065	[20]	N/A
STAR (v. 2.5.3a)	[21]	N/A
htseq-count (v. 0.9.1)	[22]	N/A
RSeQC (v. 2.3.7)	[23]	N/A
R version 4.0.2	http://cran.r-project.org/	N/A
R Bioconductor package "edgeR"	[24]	N/A
R Bioconductor package “limma”	[25]	N/A
ShinyGO v0.61:Gene Ontology Enrichment Analysis	[26]	http://bioinformatics.sdstate.edu/go/
R package “visNetwork”	http://cran.r-project.org/	N/A
R package “igraph”	http://cran.r-project.org/	N/A
ImageJ	http://rsb.info.nih.gov/ij	N/A
MS-Dial software	[27]	N/A
LOWESS/Spline normalisation program	[27]	N/A
R package “agricolae"	http://cran.r-project.org/	
ZEN 2012 v. 8,0,7273 (black edition-64 bit)	LSM 710	N/A
ImageQuant LAS 4000	GE Healthcare, Buckinghamshire,	N/A

	UK	

References

1 Hase, Y. *et al.* Ectopic endoreduplication caused by sterol alteration results in serrated petals in Arabidopsis. *J Exp Bot* **56**, 1263-1268, doi:10.1093/jxb/eri122 (2005).

2 Carland, F. M. The Identification of CVP1 Reveals a Role for Sterols in Vascular Patterning. *The Plant Cell Online* **14**, 2045-2058, doi:10.1105/tpc.003939 (2002).

3 Carland, F., Fujioka, S. & Nelson, T. The sterol methyltransferases SMT1, SMT2, and SMT3 influence Arabidopsis development through nonbrassinosteroid products. *Plant Physiol* **153**, 741-756, doi:10.1104/pp.109.152587 (2010).

4 Lorrain, S., Allen, T., Duek, P. D., Whitelam, G. C. & Fankhauser, C. Phytochrome-mediated inhibition of shade avoidance involves degradation of growth-promoting bHLH transcription factors. *Plant J* **53**, 312-323, doi:10.1111/j.1365-313X.2007.03341.x (2008).

5 Fujimori, T., Yamashino, T., Kato, T. & Mizuno, T. Circadian-controlled basic/helix-loop-helix factor, PIL6, implicated in light-signal transduction in Arabidopsis thaliana. *Plant Cell Physiol* **45**, 1078-1086, doi:10.1093/pcp/pch124 (2004).

6 Leivar, P. *et al.* The Arabidopsis phytochrome-interacting factor PIF7, together with PIF3 and PIF4, regulates responses to prolonged red light by modulating phyB levels. *Plant Cell* **20**, 337-352, doi:10.1105/tpc.107.052142 (2008).

7 de Wit, M., Ljung, K. & Fankhauser, C. Contrasting growth responses in lamina and petiole during neighbor detection depend on differential auxin responsiveness rather than different auxin levels. *New Phytol* **208**, 198-209, doi:10.1111/nph.13449 (2015).

8 Galvao, V. C. *et al.* PIF transcription factors link a neighbor threat cue to accelerated reproduction in Arabidopsis. *Nat Commun* **10**, 4005, doi:10.1038/s41467-019-11882-7 (2019).

9 Zhang, B. *et al.* BLADE-ON-PETIOLE proteins act in an E3 ubiquitin ligase complex to regulate PHYTOCHROME INTERACTING FACTOR 4 abundance. *Elife* **6**, doi:10.7554/eLife.26759 (2017).

10 Carland, F. & Nelson, T. CVP2- and CVL1-mediated phosphoinositide signaling as a regulator of the ARF GAP SFC/VAN3 in establishment of foliar vein patterns. *Plant J* **59**, 895-907, doi:10.1111/j.1365-313X.2009.03920.x (2009).

11 Brunoud, G. *et al.* A novel sensor to map auxin response and distribution at high spatio-temporal resolution. *Nature* **482**, 103-106, doi:10.1038/nature10791 (2012).

12 Hofius, D. *et al.* Autophagic components contribute to hypersensitive cell death in Arabidopsis. *Cell* **137**, 773-783, doi:10.1016/j.cell.2009.02.036 (2009).
13 Thompson, A. R., Doelling, J. H., Suttangkakul, A. & Vierstra, R. D. Autophagic Nutrient Recycling in Arabidopsis Directed by the ATG8 and ATG12 Conjugation Pathways. *Plant Physiology* **138**, 2097-2110, doi:10.1104/pp.105.060673 (2005).
14 Nozue, K. *et al.* Shade avoidance components and pathways in adult plants revealed by phenotypic profiling. *PLoS Genet* **11**, e1004953, doi:10.1371/journal.pgen.1004953 (2015).
15 Hu, S., Ye, H., Cui, Y. & Jiang, L. AtSec62 is critical for plant development and is involved in ER-phagy in Arabidopsis thaliana. *J Integr Plant Biol* **62**, 181-200, doi:10.1111/jipb.12872 (2020).
16 Stephani, M. *et al.* A cross-kingdom conserved ER-phagy receptor maintains endoplasmic reticulum homeostasis during stress. *Elife* **9**, doi:10.7554/eLife.58396 (2020).
17 Bensmihen, S. *et al.* Analysis of an activated ABI5 allele using a new selection method for transgenic Arabidopsis seeds. *FEBS Letters* **561**, 127-131, doi:10.1016/s0014-5793(04)00148-6 (2004).
18 Martin, M. Cutadapt removes adapter sequences from high-throughput sequencing reads. *EMBnet.journal* **17**, doi:10.14806/ej.17.1.200 (2011).
19 Wingett, S. W. & Andrews, S. FastQ Screen: A tool for multi-genome mapping and quality control. *F1000Res* **7**, 1338, doi:10.12688/f1000research.15931.2 (2018).
20 Davis, M. P., van Dongen, S., Abreu-Goodger, C., Bartonicek, N. & Enright, A. J. Kraken: a set of tools for quality control and analysis of high-throughput sequence data. *Methods* **63**, 41-49, doi:10.1016/j.ymeth.2013.06.027 (2013).
21 Dobin, A. *et al.* STAR: ultrafast universal RNA-seq aligner. *Bioinformatics* **29**, 15-21, doi:10.1093/bioinformatics/bts635 (2013).
22 Anders, S., Pyl, P. T. & Huber, W. HTSeq--a Python framework to work with high-throughput sequencing data. *Bioinformatics* **31**, 166-169, doi:10.1093/bioinformatics/btu638 (2015).
23 Wang, L., Wang, S. & Li, W. RSeQC: quality control of RNA-seq experiments. *Bioinformatics* **28**, 2184-2185, doi:10.1093/bioinformatics/bts635 (2012).
24 Robinson, M. D., McCarthy, D. J. & Smyth, G. K. edgeR: a Bioconductor package for differential expression analysis of digital gene expression data. *Bioinformatics* **26**, 139-140, doi:10.1093/bioinformatics/btp616 (2010).
25 Ritchie, M. E. *et al.* limma powers differential expression analyses for RNA-sequencing and microarray studies. *Nucleic Acids Res* **43**, e47, doi:10.1093/nar/gkv007 (2015).
26 Ge, S. X., Jung, D. & Yao, R. ShinyGO: a graphical gene-set enrichment tool for animals and plants. *Bioinformatics* **36**, 2628-2629, doi:10.1093/bioinformatics/btz931 (2020).

27 Tsugawa, H. *et al.* MS-DIAL: data-independent MS/MS deconvolution for comprehensive metabolome analysis. *Nat Methods* **12**, 523-526, doi:10.1038/nmeth.3393 (2015).
