# Supplementary material for: A combination of plasma membrane sterol biosynthesis and autophagy is required for shade-induced hypocotyl elongation
Source: Nat Commun. 2022 Oct 10;13:5659. doi: 10.1038/s41467-022-33384-9 (PMC9550796; doi:10.1038/s41467-022-33384-9)
Supplement: Supplementary file 13 — Reporting Summary [file 41467_2022_33384_MOESM13_ESM.pdf]

nature portfolio

Corresponding author(s): Christian Fankhauser

Last updated by author(s): 2022-9-2

Reporting Summary

Nature Portfolio wishes to improve the reproducibility of the work that we publish. This form provides structure for consistency and transparency in reporting. For further information on Nature Portfolio policies, see our Editorial Policies and the Editorial Policy Checklist.

Statistics

For all statistical analyses, confirm that the following items are present in the figure legend, table legend, main text, or Methods section.

n/a	Confirmed	
☐	☒	The exact sample size (*n*) for each experimental group/condition, given as a discrete number and unit of measurement
☐	☒	A statement on whether measurements were taken from distinct samples or whether the same sample was measured repeatedly
☐	☒	The statistical test(s) used AND whether they are one- or two-sided<br>*Only common tests should be described solely by name; describe more complex techniques in the Methods section.*
☐	☒	A description of all covariates tested
☒	☐	A description of any assumptions or corrections, such as tests of normality and adjustment for multiple comparisons
☐	☒	A full description of the statistical parameters including central tendency (e.g. means) or other basic estimates (e.g. regression coefficient) AND variation (e.g. standard deviation) or associated estimates of uncertainty (e.g. confidence intervals)
☐	☒	For null hypothesis testing, the test statistic (e.g. *F*, *t*, *r*) with confidence intervals, effect sizes, degrees of freedom and *P* value noted<br>*Give P values as exact values whenever suitable.*
☒	☐	For Bayesian analysis, information on the choice of priors and Markov chain Monte Carlo settings
☒	☐	For hierarchical and complex designs, identification of the appropriate level for tests and full reporting of outcomes
☒	☐	Estimates of effect sizes (e.g. Cohen's *d*, Pearson's *r*), indicating how they were calculated

Our web collection on statistics for biologists contains articles on many of the points above.

Software and code

Policy information about availability of computer code

Data collection	Sequence reads from FASTQ files were processed as follows: Reads that did not pass Illumina's filtering were removed. Adapters and low-quality 3' ends were trimmed from the reads using Cutadapt (v1.8) (Martin 2011). Reads matching to ribosomal RNA sequences were removed with fastq_screen (v. 0.11.1). Remaining reads were further filtered for low complexity with reaper (v. 15-065, Davis et al. 2013). Reads were aligned against Arabidopsis thaliana.TAIR10.39 genome using STAR (v. 2.5.3a, Dobin et al. 2013). The number of read counts per gene locus was summarized with htseq-count (v. 0.9.1, Anders et al. 2014) using Arabidopsis thaliana.TAIR10.39 gene annotation. Quality of the RNA-seq data alignment was assessed using RSeQC (v. 2.3.7, Wang et al. 2012).
Data analysis	Statistical analysis was performed for genes independently in R (R version 4.0.2). All steps described here were performed separately for the samples from hypocotyls and cotyledons (except for the initial clustering of all samples together). Genes with low counts were filtered out according to the rule of 1 count(s) per million (cpm) in at least 1 sample. The number of genes retained in the analyses based on this filtering is different for hypocotyls and cotyledons. Library sizes were scaled using TMM normalization. Subsequently, the normalized counts were transformed to cpm values and a log2 transformation was applied by means of the function cpm with the parameter setting prior.counts = 1 (edgeR).<br>Differential expression was computed with the R Bioconductor package "limma" by fitting data to a linear model. The approach limma-trend was used. Fold changes were computed and a moderated t-test was applied. P-values were adjusted using the Benjamini-Hochberg (BH) method, which controls for the false discovery rate (FDR), globally across several comparisons of experimental conditions.

For manuscripts utilizing custom algorithms or software that are central to the research but not yet described in published literature, software must be made available to editors and reviewers. We strongly encourage code deposition in a community repository (e.g. GitHub). See the Nature Portfolio guidelines for submitting code & software for further information.

Data

Policy information about availability of data

All manuscripts must include a data availability statement. This statement should provide the following information, where applicable:

- Accession codes, unique identifiers, or web links for publicly available datasets
- A description of any restrictions on data availability
- For clinical datasets or third party data, please ensure that the statement adheres to our policy

The RNA-Seq data is available in the following repository: NCBI GEO, accession number GSE174655. During the review process, it is accessible with reviewer token axitmakgfdklfgz. It will be made public at publication time. The MS data for lipid measurements was deposited in Metabolights (https://www.ebi.ac.uk/metabolights/). Untargeted lipidomic analysis with the unique identifier MTBLS2796, MTBLS5753 for the sphingolipidomics and MTBLS5766 for sterols.

Human research participants

Policy information about studies involving human research participants and Sex and Gender in Research.

Reporting on sex and gender	N/A
Population characteristics	N/A
Recruitment	N/A
Ethics oversight	N/A

Note that full information on the approval of the study protocol must also be provided in the manuscript.

Field-specific reporting

Please select the one below that is the best fit for your research. If you are not sure, read the appropriate sections before making your selection.

[x] Life sciences   [ ] Behavioural & social sciences   [ ] Ecological, evolutionary & environmental sciences

For a reference copy of the document with all sections, see nature.com/documents/nr-reporting-summary-flat.pdf

Life sciences study design

All studies must disclose on these points even when the disclosure is negative.

Sample size	Sample size for hypocotyl measurements are indicated in all figures. For quantified western blots we provide data from 4 biological replicas. RNA seq analysis was performed from 3 biological replicas. Lipid analysis is performed from 4 to 5 replicas.
Data exclusions	No data was excluded
Replication	Experiments were performed several times and we present one representative example. The RNA seq and lipidomic analyses are from one experiment with several biological replicas (see above). However, key findings are followed up with for example RT-qPCR experiments to confirm and extend observation from the RNA seq analysis.
Randomization	To avoid plate and position effects, we use several randomly positioned genotypes per plate and each genotype is present on more than 1 plate. Sampling order between treatments and genotypes is also randomized.
Blinding	Unknown number identities for the different genotypes/conditions were used for RNA seq and lipidomic analyses. For figure 7d, several lab members scored unlabeled pictures.

Reporting for specific materials, systems and methods

We require information from authors about some types of materials, experimental systems and methods used in many studies. Here, indicate whether each material, system or method listed is relevant to your study. If you are not sure if a list item applies to your research, read the appropriate section before selecting a response.

Materials & experimental systems

n/a	Involved in the study	
☐	☒	Antibodies
☒	☐	Eukaryotic cell lines
☒	☐	Palaeontology and archaeology
☐	☒	Animals and other organisms
☒	☐	Clinical data
☒	☐	Dual use research of concern

Methods

n/a	Involved in the study	
☒	☐	ChIP-seq
☒	☐	Flow cytometry
☒	☐	MRI-based neuroimaging

Antibodies

Antibodies used

Anti-GFP JL-8, Clontech, California, USA, Cat. No. 632380/632381
Polyclonal histone H3, Abcam, Cambridge, UK, Cat. No.1791
Anti-TUB, Abicode, California, USA, Cat. No. M0267-1a
Horseradish peroxidase (HRP)-conjugated anti-mouse, Promega, Madison, USA, Cat. No. W4011
Horseradish peroxidase (HRP)-conjugated anti-rabbit, Promega, Madison, USA, Cat. No. W4021

Validation

The antibodies were validated by the respective manufacturers

Animals and other research organisms

Policy information about studies involving animals; ARRIVE guidelines recommended for reporting animal research, and Sex and Gender in Research

Laboratory animals

Our study involved no animals but plants (Arabidopsis thaliana and Brassica rapa)

Wild animals

N/A

Reporting on sex

N/A

Field-collected samples

N/A

Ethics oversight

N/A

Note that full information on the approval of the study protocol must also be provided in the manuscript.
